# A new cave-dwelling species of *Hahnia* C. L. Koch, 1841 from Guizhou Province, China (Araneae, Hahniidae)

**DOI:** 10.3897/BDJ.11.e113400

**Published:** 2023-11-30

**Authors:** Jiahui Gan, Cheng Wang, Xiaoqi Mi

**Affiliations:** 1 Guizhou Key Laboratory of Biodiversity Conservation and Utilization in the Fanjing Mountain Region, Tongren University, Tongren, China Guizhou Key Laboratory of Biodiversity Conservation and Utilization in the Fanjing Mountain Region, Tongren University Tongren China

**Keywords:** diversity, morphology, southwest China, taxonomy

## Abstract

**Background:**

*Hahnia*, the most species-diversity genus of the comb-tailed spider family Hahniidae Bertkau, 1878, compromises 102 species distributed worldwide. To date, 24 species have been recorded from China.

**New information:**

A new species of the genus *Hahnia* C. L. Koch, 1841 is described, based on both sexes from Shanyang Cave of Guizhou Province, China and is named *H.jiangkou* **sp. nov.** Diagnostic photos of habitus and copulatory organs, as well as a distributional map, are provided.

## Introduction

*Hahnia* is the most species-rich genus of the family Hahniidae, containing 102 valid species that are distributed worldwide, with 24 species being recorded from China, including 16 endemics so far (World Spider Catalog 2023). Despite the impressive species diversity, the genus remains poorly studied because: 1) more than one-third (38) of its species are known only from a single sex (World Spider Catalog 2023) and 2) Hahnia has not been the subject of any global or regional revisions and should, at this time, be putatively considered as paraphyletic (Huang et al. 2017). Similar to most spider fauna, the taxonomy of Chinese *Hahnia* began at the latter end of the 20^th^ century, but knowledge has rapidly increased in less than four decades by a series of scattered descriptions and the systematic studies of *Hahnia* species from the Xishuangbanna and Gaoligong Mountains provided by Zhang et al. (2011) and Huang et al. (2017).

In our recent survey of caves from Tongren, Guizhou, China, a comb-tailed spider was found and has been recognised as new to science after being compared with other congeners and is described as *H.jiangkou* sp. nov. herein.

## Materials and methods

Specimens were collected by hand-collecting and were preserved in 80% ethanol for morphological study. All specimens are deposited in the Museum of Tongren University, China (TRU). The specimens were examined with an Olympus SZX10 stereomicroscope. After dissection, the vulva was cleared in trypsin enzyme solution before examination and imaging. The left male palp was used for the descriptions and illustrations. Photos of the copulatory organs and habitus were taken with a Kuy Nice CCD camera, mounted on an Olympus BX43 compound microscope. Compound focus images were generated using Helicon Focus v. 6.7.1.

All measurements are given in millimetres (mm). Leg measurements are given as total length (femur, patella, tibia, metatarsus, tarsus). References to figures in the cited papers are listed in lowercase type (fig. or figs.) and figures in this paper are noted with an initial capital (Fig. or Figs.).

Abbreviations used in the text and figures are as follows: AME anterior median eye; ALE anterior lateral eye; AR atrial ridge; At atrium; C conductor; CD copulatory duct; CF cymbial furrow; E embolus; FD fertilisation duct; MA median apophysis; MOA median ocular area; PME posterior median eye; PLE posterior median eye; RPA retrolateral patellar apophysis; RTA retrolateral tibial apophysis; S spermatheca; Ss subspermatheca.

## Taxon treatments

### Hahnia jiangkou

Gan, Wang & Mi sp. nov.

3057B03A-41B2-5113-94D1-06811E2EE494

F367C1EE-C7D4-429A-83E0-3565C595116B

#### Materials

- **Type status:** Holotype. **Occurrence:** sex: male; occurrenceID: 5194CA23-772E-5AF5-85EA-C3890C30CFD3; **Location:** country: China; stateProvince: Guizhou; county: Jiangkou; locality: Minxiao Township, Shanyang Cave; verbatimElevation: 784 m; verbatimLatitude: 27°41′1.6″N; verbatimLongitude: 108°36′48.6″E; **Identification:** identifiedBy: Jiahui Gan; **Event:** samplingProtocol: by hand; year: 2023; month: 3; day: 12; habitat: cave
- **Type status:** Paratype. **Occurrence:** individualCount: 2; sex: females; occurrenceID: 9DF11921-5D69-5084-9A5B-33B949512893; **Location:** country: China; stateProvince: Guizhou; county: Jiangkou; locality: Minxiao Township, Shanyang Cave; verbatimElevation: 784 m; verbatimLatitude: 27°41′1.6″N; verbatimLongitude: 108°36′48.6″E; **Identification:** identifiedBy: Jiahui Gan; **Event:** samplingProtocol: by hand; year: 2023; month: 3; day: 12; habitat: cave
- **Type status:** Paratype. **Occurrence:** individualCount: 4; sex: females; occurrenceID: BD0E595D-996E-53D6-9237-E34DFDF07C87; **Location:** country: China; stateProvince: Guizhou; county: Jiangkou; locality: Minxiao Township, Shanyang Cave; verbatimElevation: 784 m; verbatimLatitude: 27°41′1.6″N; verbatimLongitude: 108°36′48.6″E; **Identification:** identifiedBy: Jiahui Gan; **Event:** samplingProtocol: by hand; year: 2022; month: 4; day: 26; habitat: cave

#### Description

**Figure 1.**
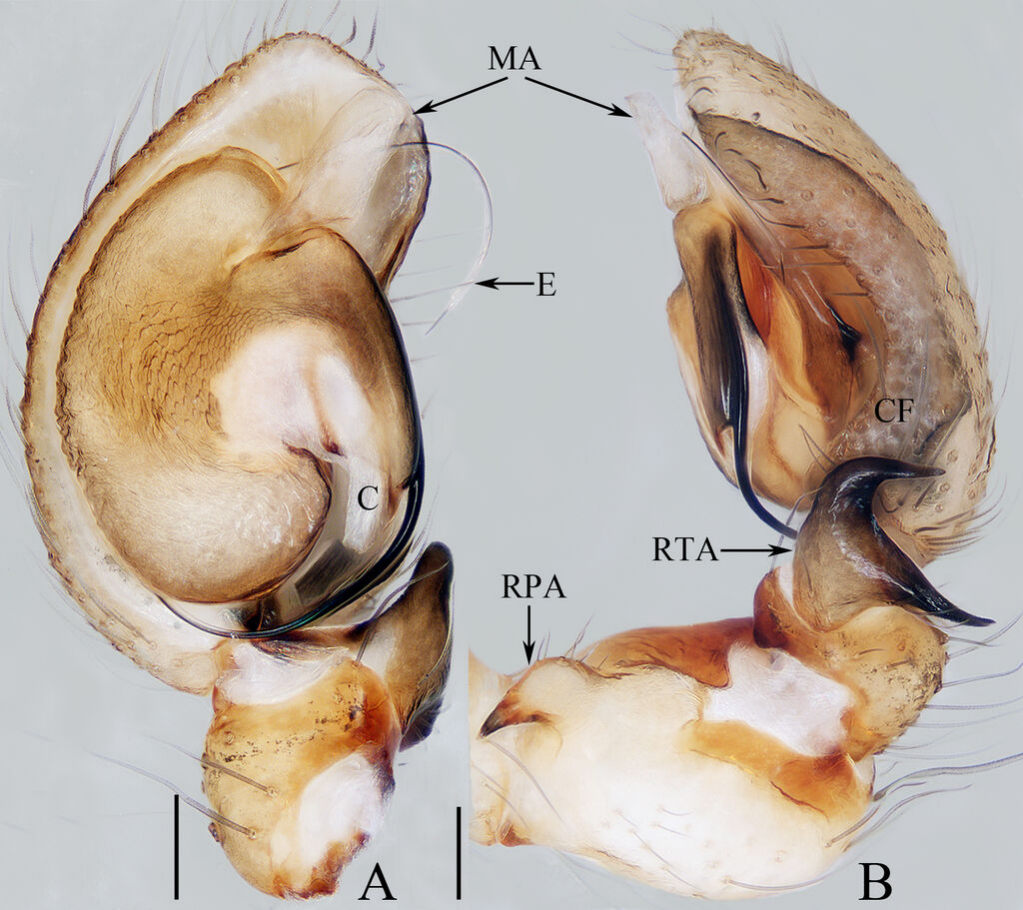
Male palp of *Hahniajiangkou* sp. nov., holotype. **A** ventral; **B** retrolateral. Scale bars: 0.1 mm. Abbreviations：C conductor; CF cymbial furrow; E embolus; MA median apophysis; RPA retrolateral patellar apophysis; RTA retrolateral tibial apophysis.

**Figure 2.**
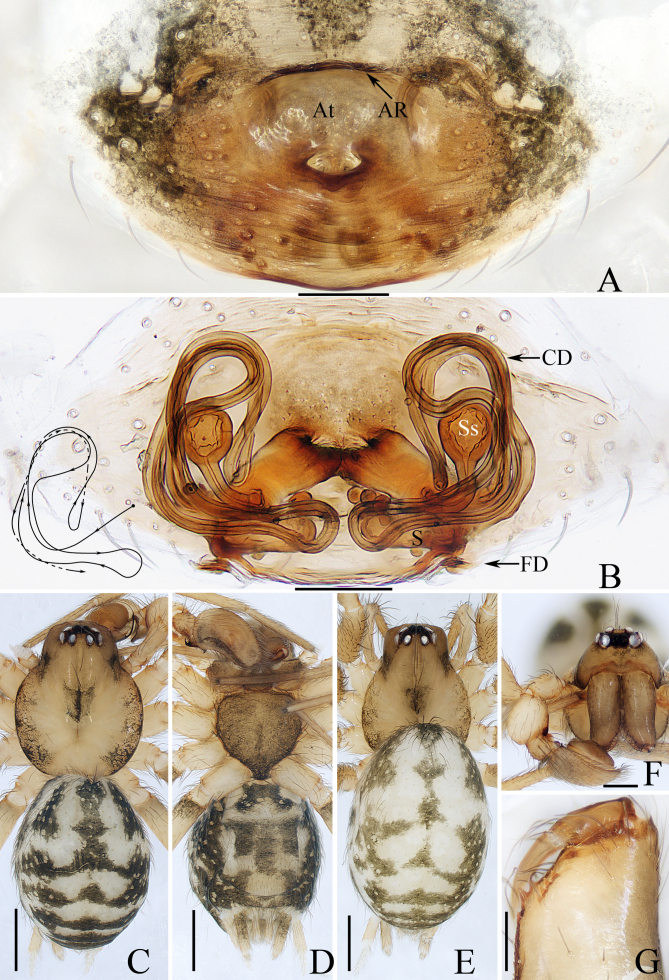
*Hahniajiangkou* sp. nov. female paratype and male holotype. **A** epigyne, ventral; **B** vulva, dorsal; **C** male habitus, dorsal; **D** ditto, ventral; **E** female habitus, dorsal; **F** male carapace, frontal; **G** male chelicera, posterior. Scale bars: A, B, G (0.1 mm); C–E (0.5 mm); F (0.2 mm). Abbreviations: AR atrial ridge; At atrium; CD copulatory duct; FD fertilisation duct; S spermatheca; Ss subspermatheca.

**Male** (holotype, Fig. 1, Fig. 2C, D, F and G): Total length 2.65. Carapace 1.26 long, 1.03 wide. Abdomen 1.41 long, 1.08 wide. Eye sizes: AME 0.06, ALE 0.08, PME 0.06, PLE 0.08. Distance between eyes: AME–AME 0.08, AME–ALE 0.08, PME–PME 0.14, PME–PLE 0.09, ALE–PLE 0.09. MOA 0.17 long, front width 0.12, back width 0.21. Clypeus 0.15 high. Leg measurements: I 4.22 (1.20, 0.38, 1.03, 0.88, 0.73); II 3.99 (1.18, 0.33, 0.95, 0.85, 0.68); III 3.62 (1.00, 0.33, 0.83, 0.83, 0.63); IV 4.42 (1.20, 0.38, 1.08, 1.03, 0.73). Carapace (Fig. 2C and F) pale yellow to yellow, with elevated cephalon and almost round, flat thorax bearing brown markings anterior to fovea and irregular, lateromarginal, brown markings; fovea red-brown, longitudinal. Chelicerae (Fig. 2F and G) yellow, both margins with three teeth. Endites lighter than chelicerae, covered with sparse brown setae. Labium darker than endites. Sternum dark brown, almost heart-shaped, covered with dark setae. Legs pale to yellow. Abdomen (Fig. 2C and D) sub-oval, dorsum grey pale to dark brown, with a pair of longitudinal, grey pale, anteromedian stripes separated by the longitudinal, dark brown stripe and alternate grey pale and dark brown transverse stripes medioposteriorly; venter pale to dark brown, covered with short thin setae.

Palp (Fig. 1A and B): patella enlarged, almost as long as wide in retrolateral view; RPA proximally located, curved towards the dorsal side at the base and then tapering to a pointed tip; tibia short, with the sclerotised RTA bifurcated into two rami extending dorsally and with pointed tips; cymbium rather flat, with a retrolateral furrow about five-sixths its length; bulb flat, almost oval; embolus enlarged at base, followed by the remainder of the flagelliform originating at ca. 3:30 o’clock position and extending almost to a circle; conductor membranous, slightly longer than wide, close to the origin of the flagelliform portion of the embolus; MA membranous, sheet-shaped, almost quadrate, located anteriorly to the base of embolus, extending antero-retrolaterally.

**Female** (paratype, Fig. 2A, B and E): Total length 2.83. Carapace 1.20 long, 0.98 wide. Abdomen 1.90 long, 1.30 wide. Clypeus 0.14 high. Eye sizes: AME 0.05, ALE 0.09, PME 0.10, PLE 0.10. Distance between eyes: AME–AME 0.08, AME–ALE 0.09, PME–PME 0.16, PME–PLE 0.10, ALE–PLE 0.10. MOA 0.19 long, front width 0.13, back width 0.24. Leg measurements: I 3.87 (1.13, 0.40, 0.93, 0.78, 0.63); II 3.66 (1.10, 0.38, 0.85, 0.73, 0.60); III 3.41 (0.95, 0.35, 0.78, 0.75, 0.58); IV 4.41 (1.18, 0.40, 1.13, 1.00, 0.70). Habitus (Fig. 2E) similar to that of male, except paler.

Epigyne (Fig. 2A and B): slightly wider than long; atrium oval, deeply concave, anteromedially located, with an almost straight anterior ridge; copulatory openings fused, located at the posterior portion of atrium; copulatory ducts widened and flat at the base and then acutely narrowed into tube-shaped portions, which form complicated paths; subspermathecae spherical, connected to the base of copulatory ducts; spermathecae elongated, about two times as long as wide; fertilisation ducts originating from the outer-posterior margins of spermathecae, bilaterally extended.

#### Diagnosis

The male of *Hahniajiangkou* sp. nov. can be easily distinguished from other congeners by the bifurcated RTA (Fig. 1B), whereas it is not bifurcated and generally semicircular in others. The female resembles that of *H.weiningensis* Huang, Chen & Zhang, 2018 in the general shape of the epigyne, especially the basally widened copulatory ducts, but it can be easily distinguished by the following: 1) the copulatory openings are medially located (Fig. 2A) versus posteriorly located in *H.weiningensis* (Huang et al. 2018: figs. 8 and 13); 2) the spermathecae are almost quadrate (Fig. 2B) versus almost pyriform in *H.weiningensis* (Huang et al. 2018: figs. 9 and 14); 3) the copulatory ducts are extending posterolaterally at base (Fig. 2B) versus extending anterolaterally in *H.weiningensis* (Huang et al. 2018: figs. 9 and 14).

#### Etymology

The specific name derives from the type locality: Jiangkou County; noun in apposition.

#### Distribution

**Figure 3.**
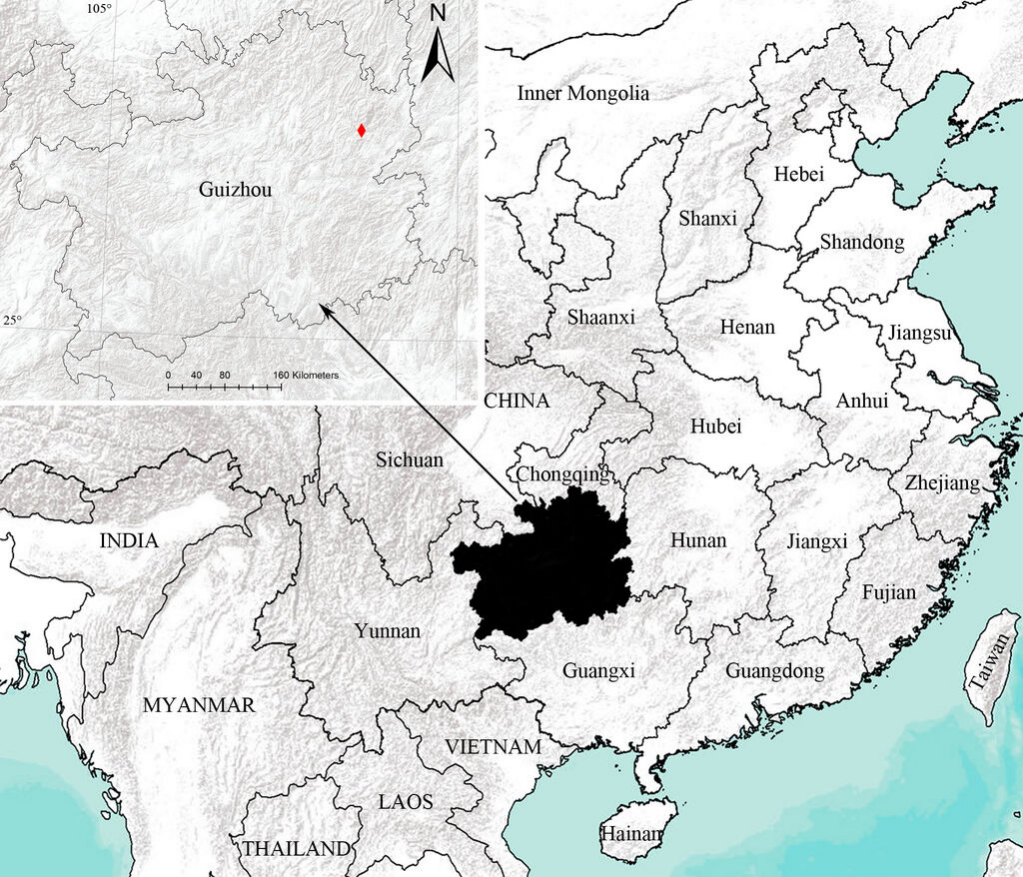
The type locality of *Hahniajiangkou* sp. nov.

Known only from the type locality in Guizhou, China (Fig. 3).

#### Taxon discussion

The species is placed into *Hahnia* due to its sharing a series of consistent characters with other congeners, such as the presence of patellar apophysis, membranous median apophysis, cymbial furrow and filiform embolus originating retrolaterally, as well as the presence of spherical subspermathecae. However, it is worth mentioning that the species is also unique for the bifurcated RTA, the absence of an epigynal hood (vs. semicircular RTA, with a pair of hoods generally located behind the epigastric furrow in others), all of which indicate that its generic position may need further confirmation.

## Supplementary Material

XML Treatment for Hahnia jiangkou
